# Demographic Associations with GPS-Inferred Routine Activity Spaces: Data from the Everyday Environments and Experiences (E3) Study

**DOI:** 10.3390/s26061902

**Published:** 2026-03-18

**Authors:** Nathan Ryder, Ulf G. Bronas, Jason Westra, Jieqi Tu, Evan De Jong, Yosef Bodovski, Kiarri N. Kershaw, Nathan L. Tintle

**Affiliations:** 1 Department of Population Health Nursing Science, College of Nursing, University of Illinois Chicago, Chicago, IL 60612, USA; 2 Division of Research and Scholarship, School of Nursing, Columbia University, New York, NY 10032, USA; ub2154@cumc.columbia.edu; 3 Department of Biostatistics, School of Public Health, University of Illinois Chicago, Chicago, IL 60612, USA; 4 Population Research Institute, Pennsylvania State University, University Park, PA 16802, USA; 5 Department of Preventive Medicine, Northwestern University Feinberg School of Medicine, Chicago, IL 60611, USA

**Keywords:** exercise, physical activity, urban, systemic inequity

## Abstract

People in midlife interact with several different environments during their daily life including employment, leisure, commuting, and various family responsibilities, a concept defined as activity space. However, little is known about how these activity spaces contribute to individuals’ daily health behavior choices. The Everyday Environments and Experiences (E3) study was conducted to explore these relationships. In this paper, we provide a reproducible GPS processing workflow to generate time-weighted exposure measures (activity spaces) inferred from 21 days of continuous GPS monitoring among 340 midlife adults in Cook County, Illinois (*n* = 340) from the E3 study. Data from waist-mounted GPS devices that recorded one-minute location epochs were aggregated after excluding time spent within an 800 m buffer around the home. For each epoch, we derived proximity and kernel density measures for eleven food and physical-activity-related location types (e.g., supermarkets, fitness facilities), along with twenty-six environmental context variables (e.g., land use, crime, population density). Time-weighted averages characterized each participant’s typical non-home environmental exposure. After adjustment for environmental context, age and gender were generally unrelated to activity-space measures. However, Black and Hispanic participants (as compared to White participants) spent less time near both food and physical-activity resources, suggesting systemic inequities in access beyond neighborhood composition. These findings highlight the need to move beyond static residential measures toward time-weighted, dynamic assessments of environmental exposure. They also indicate that racial and ethnic disparities in routine activity space may reflect structural inequities shaping daily physical activity and access to healthy food. Future research is needed to explore how these observed disparities translate into differences into disease risk, using longer exposure periods and different geographic settings to identify causal pathways and inform multi-level interventions.

## 1. Introduction

Unhealthy dietary and physical activity (PA) behaviors contribute to an increased risk for obesity, cancer, and other aging-related chronic diseases [1,2,3]. These behaviors further accentuate health disparities [4], especially during the transitional period of midlife. Midlife is a known vulnerable life stage when obesity rates peak and chronic diseases emerge, often related to modifiable lifestyle risk factors [5,6]. Residential neighborhood environments provide opportunities, barriers, and cues/triggers to engage in healthy or unhealthy behaviors, making them a focus of built environment public health research. Interventions and policies that impact neighborhood-built environment on public health are a major focus of Healthy People 2030 [7]. However, research findings on residential neighborhood environment–behavior associations are inconsistent, and effect sizes are small [8]. Built environment–physical activity research has mixed findings, potentially due, at least in part, to heterogeneity in exposure definitions and reliance on cross-sectional, home-centric measures [9,10]. One potential explanation is that additional environmental exposures during daily living contribute to levels of healthy behaviors. Much of the current research on residential neighborhoods does not consider the activities of daily living in midlife.

People in midlife interact with several different environments during their daily life, including employment, leisure, commuting, and various family responsibilities, a concept defined as activity space [11]. Failure to consider these activity spaces (the full daily exposure to various environments) may cause research to date to miss the full impact of environmental differences on health behaviors. Activity space research has increasingly leveraged passive mobility data (e.g., GPS) to capture real-world exposures to food and built environments [12]. Research comparing residence-based versus mobility-based exposures suggests that home-centric definitions can substantially differ from exposures observed across daily travel [13]. Quantifying aspects of an individual’s activity space is necessary to understand whether activity spaces do in fact impact health behavior, and how these explanations may vary based on other individual and environmental factors. To date, little is known about the putative impact of activity space on health behavior and how best to measure and quantify activity spaces.

Although a growing literature uses GPS-derived activity spaces, studies vary widely in how activity spaces are constructed (e.g., buffered tracks, convex hulls, ellipses) and in which exposure metrics are derived: limiting comparability and consensus on best practices [12]. For example, one option uses activity locations from raw trajectories and applies clustering algorithms, such as a kernel-based approach, which detects locations where individuals remain for a minimum time and distance threshold [14]. Alternatively, density-based hotspot methods such as kernel density estimation (KDE) aggregate GPS points or stay locations to estimate continuous spatial intensity surfaces that highlight frequently visited areas [15]. Another option (e.g., DBSCAN) uses clustering by grouping nearby GPS observations into discrete hotspots without assuming a parametric distribution [16]. Another approach represents the overall geographic extent of movement rather than specific hotspots by computing activity space measures such as the Minimum Convex Polygon (MCP), Standard Deviation Ellipse (SDE), or Daily Path Area (DPA), which summarize the spatial footprint of an individual’s mobility [17].

The purpose of the Everyday Environment and Experiences (E3) study is to provide methodological innovation on how to measure and quantify activity spaces, while investigating how activity spaces may explain participant variations in diet and PA during midlife [8]. To do this, enrolled E3 participants wore global positioning system (GPS) tracking devices for 21 continuous days. In this manuscript, we detail a novel, reproducible, and robust method for extracting and summarizing GPS-inferred position information on participants into summaries of activity space. Activity-space-inferred data includes availability of healthy and unhealthy foods, walkability, and recreational resource availability (e.g., parks, fitness facilities, greenness). We illustrate how these activity space measures differ across participant characteristics, suggesting potentially generalizable conclusions about gender, age, and race/ethnicity differences associated with different lived experiences as quantified by activity space data. We also show how these relationships may be explained by other characteristics of the activity space (e.g., crime, poverty, population density).

## 2. Materials and Methods

### 2.1. Study

The full details of participant recruiting and participation in E3 are described elsewhere [18], but we summarize them briefly here. E3 was a cross-sectional study designed to investigate the impact of neighborhood and activity-space/environment on diet and physical activity among midlife adults residing in Cook County, Illinois. Cook County is the second most populous US county (~5.2 million residents), the eleventh most diverse county in the US (>50% of residents are Black or Hispanic), and covers approximately 945 sq miles with a population density of ~5500 residents/sq mile. Individuals eligible for this study met the following criteria: (1) self-reported as non-Hispanic (NH) White, NH Black, or Hispanic/Latino; (2) aged 40 to 64 years; (3) resided in Cook County, Illinois; (4) had access to a smartphone; and (5) provided informed consent. Participants were recruited using a variety of community-focused recruiting methods. We screened 1307 participants for inclusion, 686 of whom met an exclusion criterion (7 were uninterested). Of the 614 that met the inclusion criteria and were interested, 204 did not complete the baseline assessment, and 10 withdrew within 5 days of baseline assessment, ultimately resulting in an enrolled sample of 400 participants. Interested participants first underwent a recruitment call with a staff member to learn about what the study entails and ensure eligibility. The study was approved by the University of Illinois Chicago Institutional Review Board (IRB 2019-0630), and informed consent (e-consent) was obtained prior to study procedures.

### 2.2. GPS Protocol and Data Collection

As discussed in Bronas et al. 2026 [18], after enrollment, data collection for the E3 study occurred in three stages: a baseline visit, the three-week (21-day) study period, and a follow-up visit, all of which were performed remotely. Twenty-one days is sufficient to capture routine activity space information [19,20]. Prior to the baseline visit, each enrolled participant received a Global Positioning System (GPS) device by mail. At the remote baseline visit, participants completed short interviews and were trained/prepared for all components of data collection, including training on how to use and wear the GPS unit. After 21 days, participants ceased data collection and had a remote follow-up visit. The GPS device was then shipped back to the main study location, where participant data was downloaded to a local computer and cataloged.

The GPS units participants received were Qstarz BT-Q1000XT Bluetooth Data Logger Receivers (Qstarz, Taipei, Taiwan). Participants were instructed to wear the small (1” × 2” × 3”), lightweight, GPS unit around their waist, positioned on the front or lateral hip via a hook-and-loop fastened waistband for the entire 21-day study period. Each GPS unit was set to measure participant position at 1 min epochs. Participants were instructed to always wear the waistband except when swimming, bathing, or sleeping, and to charge the device with the provided charger when it was removed for sleeping. The Qstarz device automatically enters sleep mode to conserve battery life after 10 min of no activity. Some modest non-compliance with these instructions was noted over the course of the study, including failure to wear the device at home, when going out, or while working, and neglecting to charge the device overnight, then charging during the day when the device should be worn (the typical battery life is 42 h). In one case, a participant’s GPS device was not functioning properly and needed to be replaced. In sum, of the 400 participants enrolled in the E3 study, 362 GPS records were retrieved from returned devices, 347 of which had measurable data during the 21-day study period.

Raw data from the 347 participants was stored in comma-separated value (CSV) files, including latitude and longitude coordinates with a date and time stamp for each minute (epoch) the device was monitoring. Using R (version 4.2.0; R Core Team 2022), we truncated the raw CSV files to the study time frame (baseline visit date to 21 days) and used the AGPSR package Ver 1.0 [21] (Step 2) to clean and impute the coordinate data. Specifically, using AGPSR, we removed any location epoch that implied a speed of >130 km/h from the previous epoch. We also removed epochs outside of a pre-specified set of fourteen counties covering the Chicago metro area, (Illinois: Cook, Dekalb, Du Page, Grundy, Kane, Kendall, Lake, McHenry; Indiana: Will, Jasper, Lake, Newton, Porter; Wisconsin: Kenosha), allowing 7 min after a participant left the area before starting to drop data points. Accordingly, any epochs suggesting a position in Lake Michigan were removed 7 min after the last time the participant was positioned in an appropriate county. We then linearly interpolated any sequence of 5 or fewer missing points (i.e., 5 or fewer minutes) between two observed points. Gaps of more than 5 min were left as missing. No filtering was done on measures of GIS signal quality (e.g., DOP measures), aside from what is indicated above. After applying the AGPSR function for data cleaning and imputation, we also removed data for 7 participants who had no record of their GPS device traveling farther than 800 m from their home address by street network. Finally, we removed data for any participant on a day when they registered less than 10 h of wear time.

### 2.3. GPS Inferred Activity Space Measures

**Figure 1:**
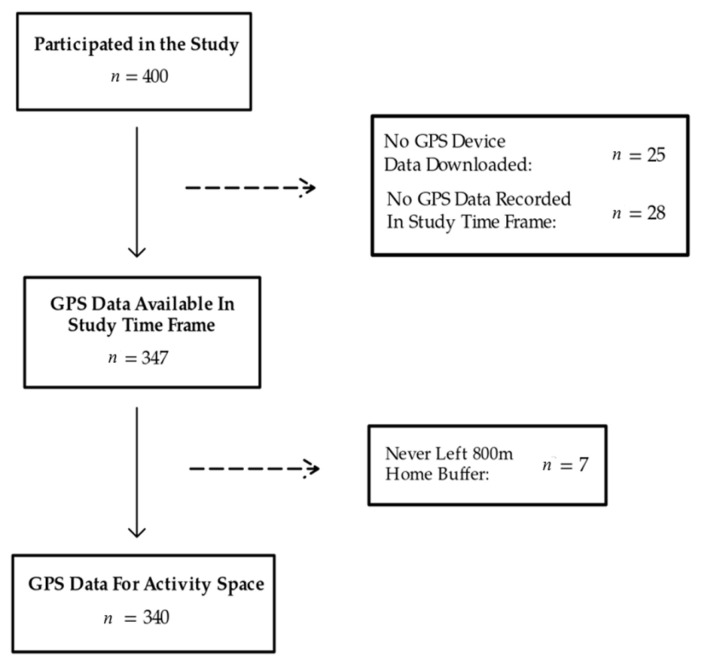
CONSORT diagram.

**Figure 2:**
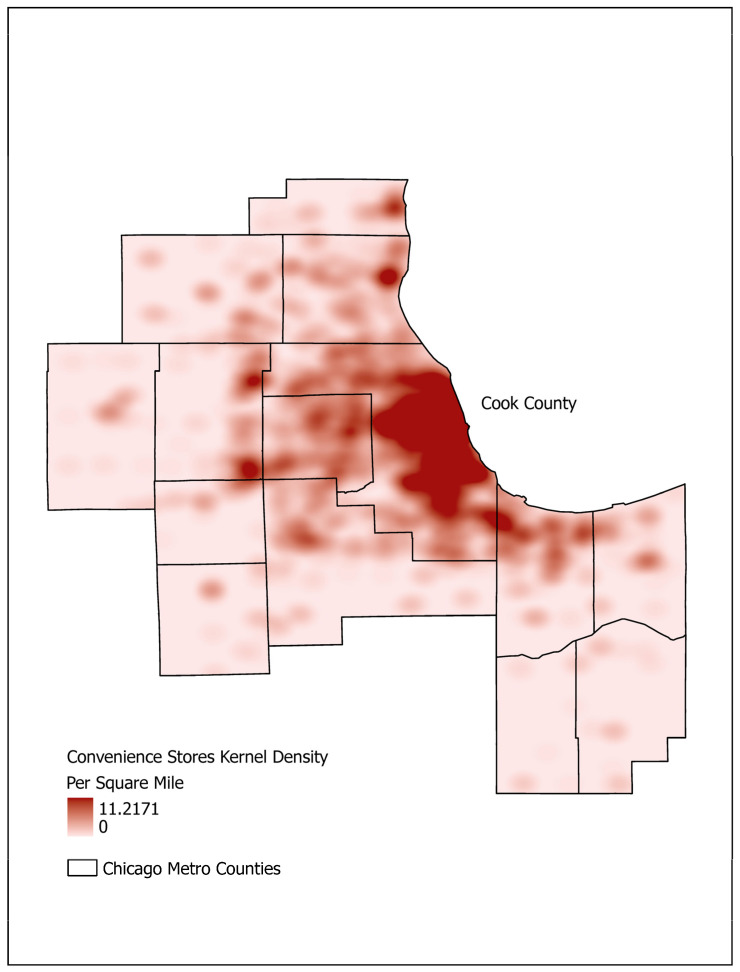
Example computation of kernel density for convenience stores.

Using the remaining 340 GPS records (Figure 1), we provided geographical context to every epoch via the ESRI GIS processing tool and database ArcGIS (Pro version: 3.1.3). To prepare the GPS data and apply the ArcGIS tools, we implemented the ArcPy package in Python (version 3.6.9). We first geocoded the participants’ home addresses to GPS coordinates, then defined an 800 m buffer around each home location by street network, meaning the resulting polygon had at most a distance of 800 m via roadway from the edge to the center location. An eight-hundred-meter buffer has been used by others and has been shown to have little impact on results as compared to other buffer sizes (e.g., 400 m or 1600 m) [22,23]. For each remaining epoch, we computed the closest distances to eleven location types (public park, bike path segment, physical activity location, pedestrian segment, public transit, street intersection, convenience store, any fast food restaurant, fast food chain restaurant, supermarket, and wholesale club store). We also computed kernel densities (per square mile) for each epoch for the same eleven location types [7]. Kernel density surfaces were fit for each location type across the Chicago metro area using the ESRI Spatial Analyst Toolbox (Version 3.1), which implements a quartic kernel function [24] (Projection: Transverse Mercator, Scale Factor 0.999975, Kernel Shape Quartic, Output unit Square mile, Equal weighting). Figure 2 illustrates the kernel density surface estimate for convenience stores for the fourteen counties included in this study. Casually, Figure 2 illustrates how dense convenience stores are at any given location across the fourteen-county area (darker red = more dense), allowing us to obtain a kernel density value for convenience stores for each epoch for each participant. The kernel density value of an epoch is calculated for a 100 m raster cell from an overall grid. Thus, we created a total of 22 separate measures of activity space (11 distance measures; 11 kernel densities (per square mile)) for each epoch. Figure 2 also shows Cook County (home county for all participants), along with the additional 13 surrounding counties where participants’ movements were captured during the study period.

### 2.4. GPS Inferred Environmental Context Measures

We also obtained twenty-six additional environmental context measures for each epoch. These were based on satellite measurements (100 m cells) of land use (two: commercial (%), entropy (%)), the normalized difference vegetative index (NDVI; two measures: January and June 2020), interpolated estimates of aggregate reported crime based on data from applied geographic solutions (eight measures: counts of burglary, murder, personal, robbery, vehicle, larceny, rape and total crime) [7], and fourteen census-tract based measures from the American Community Survey (ACS) data (Hispanic (%), NH White (%), NH Black (%), NH Asian (%), Race Entropy, Female-Headed households (%), Renter Occupied (%), Same house as 1-year ago (%), Moved within 1-year (%), Public Assistance (%), Poverty (%), Unemployment (%), Population Density and Total Population). These were all evaluated as values from 100 m raster cells in an overall grid [7].

### 2.5. Aggregated GPS Inferred Measures

To allow for an aggregate view of activity space across participants, we first averaged the epoch-level values of each participant for GPS-inferred activity space and environmental context across the entire study period after removing all time in the home buffer (800 m from home address). Thus, the resulting GPS inferred measures represent the average (typical) activity space and environmental context for each participant during the study period. Measures are inherently time-weighted to quantify exposure to food and physical activity locations, and time in different environmental contexts, because they average each 1 min epoch, meaning that the longer a participant spends at a location, the more heavily it will count in the aggregated measure.

### 2.6. Measures of Participant Characteristics

Participants self-reported a variety of demographic information including gender (male, female, other), age (years), height and weight (from which we inferred BMI as kg/m^2^), race/ethnicity (non-Hispanic White, non-Hispanic Black and Hispanic/Latino), education (less than high school, high school, some college (2–4 y) or more than 4 yrs of college), household income (<$50,000/yr, $50,000–150,000/yr, More than $150,000/yr), marital status (never married, married, divorced/separated, widowed) and Walk Score^®^ at home address. The Walk Score^®^ is a combined and standardized measure of walking distances to various key amenities [25]. We also classified each participants 21-day data collection period to its meteorological season based on: Spring (March–May), Summer (June–August), Fall (September–November) or Winter (December–February).

### 2.7. Statistical Analysis

Participant characteristics were summarized by frequencies and mean/standard deviation (SD). Two-sample *t*-tests and correlation coefficients were used to investigate bi-variate relationships. Partial correlation models with multiple covariates were used to account for environmental predictors using the ppcor package (Version 1.1) in R, in order to provide covariate adjusted estimates of Pearson correlations. Covariates included demographic and geographical context variable as specified in table footnotes. Standard consideration of outliers and normality checks were performed. Given the exploratory nature of the study, all analyses used a 0.05 significance level with two-sided tests.

## 3. Results

### 3.1. Participant Characteristics

Table 1 summarizes the characteristics of the 340 study participants with available GPS data. The sample was approximately 60% female, had a mean age of 50, and was approximately one-third non-Hispanic White, one-third non-Hispanic Black, and one-third Hispanic. The average BMI of the sample was 30, 30% had a high school education or less, and approximately 40% of the sample was currently married, compared to 34% never married, and 26% formerly married. The average Walk Score^®^ at a participant’s home address was 75. Demographics generally followed those of the city of Chicago [7], except for age, which was older by design. Furthermore, participant data were gathered fairly uniformly throughout the year (Spring: 73 participants; Summer: 84 participants; Fall: 90 participants; Winter: 93 participants).

### 3.2. GPS Data Availability

Table 2 provides summary statistics across participants for the total number of GPS points measured in the 21-day study period, the number of different days the GPS was used, latitude, and longitude. On average, data were available for 14 of the 21-day study period after excluding an 800 m zone around the participant’s home (the “home buffer”). We note that the theoretical maximum number of GPS points available for any participant is 30,240 points (21 days × 24 h × 60 min; 1 min epochs). Most participants had non-wear time, time that the GPS was turned off/charging, or days with less than 10 h of wear (excluded). For the remainder of this study, we consider activity space measures from only the GPS data observed outside of the home buffer. On average, we have ~5 h of wear time per day, per participant, for 14 days, during which time participants were outside of the home buffer. However, there was wide variability (high SD) across participants as reflected in Table 2.

### 3.3. Distribution of GPS Inferred Measurements Across Participants

Table 3 provides the distribution of participants’ average activity space measures for each of the eleven location types, including measurements for both closest distance and kernel density (per square mile) raster values. Wholesale club stores were on average the farthest away from participant locations, while street intersections were on average within 100 m of most participants. Similarly, street intersections had the highest kernel density on average, and wholesale club stores had the least. The inverse relationships between distance and density measures of a location type are displayed in a correlation heatmap in Supplementary Figure S1, as well as the positive correlations within all density measures and within all distance measures. Table 4 provides similar summary statistics for the twenty-six environmental values, with a corresponding correlation heatmap for these variables in Supplementary Figure S2.

### 3.4. Unadjusted Demographic Associations with Activity Space Measures

We calculated sample mean differences and 95% confidence intervals for the eleven kernel density activity space measures by self-reported gender, race/ethnicity, and age (Table 5). Since activity space measures are all positively correlated with each other, we observe similar unadjusted relationships for each of the density averages across each of the three demographic variables. Specifically, men tended to have higher time-weighted average densities as compared to women, meaning that men tended to be closer to food and physical activity-related locations. Similarly, average kernel densities were higher in <50-year-olds as compared to those 50 or older and were also higher in non-Hispanic White participants as compared to non-Hispanic Black or Hispanic participants. Differences between NH Black and NH White participants were all statistically significant (*p* < 0.001) for all location types. In a complementary analysis, we estimated correlations and 95% confidence intervals of the location-type kernel densities with demographics (binary indicators) (Supplementary Table S1). Supplementary Table S2 provides within-group kernel density values corresponding to Table 5. Supplementary Table S3 shows similar values by season, with no statistical evidence of differences.

### 3.5. Correlations Between Activity Space and Measures of Environmental Context

In Table 6, we provide correlations and 95% confidence intervals for activity space kernel densities and select measures of environmental context, along with color coding to illustrate correlation strength. Population density, total crime, commercial land use, and walkability all tended to be correlated with higher average kernel densities of locations, while poverty prevalence and the vegetative index were correlated with lower densities. In one exception, public transit is positively correlated with poverty prevalence when most other location types’ kernel densities were negatively correlated.

### 3.6. Adjusted Associations Between Location Type and Both Participant Demographic and Other Routine Activity Space Measures

In Table 7, we report the results of a partial correlation analysis, where for each location kernel density, we estimated the partial correlations adjusted for all the demographic variables in Table 5 and the environmental context measures from Table 6. Not all correlations remained statistically significant after accounting for other variables. Those that did remain statistically significant generally matched the direction of their unadjusted versions, though in some cases the strength of association was somewhat mediated. Age and gender were generally unrelated to location type and routine activity space, but race/ethnicity was, even after accounting for other geographic factors. In general, Black and Hispanic participants spent less time near activity locations compared to White participants. Furthermore, Black participants also spent less time near food locations, including fast food, convenience stores, and supermarkets. Most location types were associated with most other geographic variables, with food and activity locations more common in areas that were more densely populated, had higher crime levels, had higher commercial land use, and had higher walkability. On the other hand, food and activity locations were less common in areas with higher vegetation and poverty.

### 3.7. Sensitivity Analyses

We also conducted a series of sensitivity analyses where the time the participant spent within their home buffer was not excluded. These results are provided in Supplementary Tables S4, S5, and S6, which parallel Table 5, Table 6, and Table 7, respectively. Patterns of relationships are largely the same.

## 4. Discussion

This study has provided first-of-its-kind data about the routine activity spaces of participants living in an urban county in Chicago, Illinois, USA. Midlife participants in the study showed that time spent near activity and food locations differed little by age or gender, after accounting for other factors, but showed strong associations with race/ethnicity, and other geographic factors (e.g., population density, crime, poverty, and walkability indices).

Methodologically, this paper illustrates an approach to quantify characteristics of a participant’s routine activity space with regard to proximity to food and physical activity locations, based on continuously worn GPS data. Important steps in our proposed approach include careful data cleaning and computation of activity space and environmental context values across all measured time points, to allow for a time-weighted, aggregated measure. Thus, aggregated activity space and environmental context measures can be thought of as ‘exposure time’ relative to specific types of food or physical activity locations, or in different environmental contexts during the study. Exclusion of the home buffers allows for generalizability and consideration of the impact of where individuals spend their time beyond home. While home locations clearly have an important impact on participant health behavior, the inclusion of time at home overly weights the impact of the home and home neighborhood on health, and does not allow for the unique consideration of where else participants spend their time (e.g., work, social activities) and the impact of those environments on health behaviors, as we have done here. Future analyses are needed to better understand the relative contribution of home neighborhood relative to non-home activity space on health behaviors in the E3 study.

Here, we demonstrate a method of processing GPS data that provides a time-weighted view of a participant’s proximity to food and physical activity resources by leveraging a density-based hotspot approach, summarized over time. This method has the advantage of providing a non-parametric view of participant behavior, which further addresses a common critique of kernel density approaches—that they typically ignore temporal information. While promising, further work is needed to more directly compare this approach to others that have been proposed in terms of sensitivity of detecting relationships with geographic, physical activity, and food resources (e.g., dbscan; convex hull).

The apparent systemic differences have been reported previously in neighborhood-level factor studies with NH-Black and Hispanic populations having less access to healthy foods and safe physical activity environments [26]. Our study extends these previous findings to include not only neighborhood-level factors, but also the daily activity space areas outside of the home neighborhood, with less time spent near physical activity locations, suggesting that differential exposure to the built environment extends well beyond that of the home neighborhood. In contrast to neighborhood studies that generally report proximity to fast-food and convenience stores in minority populations [26], we found that Black participants spent less time during their daily living near any food locations. This finding held up even after adjustment for population density, poverty, crime, and neighborhood walkability (walk score), suggesting a systemic difference in proximity to food and physical activity across the entire community and not only at the neighborhood level. This is important when developing complex multi-level interventions that address both the local community and the broad societal levels that target structural and systemic differences. Additionally, findings show that NH-Black participants spent less time near any food location or physical activity location compared to White and Hispanic participants. This could help provide insights into why a neighborhood-level study reported poorer diet quality in Black participants, compared to both Hispanic and White participants [27]. These findings need to be further explored in other samples and environments.

In addition to race/ethnicity differences, areas of high population density, higher crime, higher commercial land use, and higher walkability were more strongly associated with proximity to food and physical activity locations. In contrast, areas of high poverty and low vegetation tended to have lower proximity to food and physical activity locations. While these patterns are generally expected, they do suggest that future analyses of this data account for environmental context when considering relationships between activity space and health behaviors. The relationship between proximity to physical activity spaces, such as parks and bike paths, and crime is inconclusive in the literature [28]. Although studies have found reduced crime rates around buildings with more public green space [29] other studies show significantly higher crime rates in and around public parks [30]. It is possible that these conflicting findings are due to investigating a limited area, or a single location/city, and how a public green space is defined (e.g., a public park, grass space, tree canopy). Additionally, given the association between crime, population density, and commercial land use, it may be necessary to control for these factors when investigating the crime rate with proximity to public green spaces. It is further likely that the extent to which a green space is used influences this relationship [28]. Similarly, the proximity between food locations and crime rate has been reported previously, especially types of food locations with fast-food [31] and supercenters [32] and crime being associated, whereas food locations such as farmers’ markets tended to be associated with lower crime rates [33]. Our findings are generally supportive and extend these previous findings. Future analyses will explore these relationships in depth as they are related to physical activity and healthy eating.

Despite the strengths of the study, there are limitations worth acknowledging. Patterns of race/ethnic disparity, while striking and beyond those accounted for by other geographic variables, gender, or age, may still be explained by a variety of other factors not considered here. While recruiting of participants took a multi-faceted approach, most participants were enrolled via subway flyers, which led to a sample that tended to be of slightly higher education and income as compared to the average Chicagoan. While the period of the study was 21 days, not all participants had complete data across all 21 days. However, the average number of days of data was 14 days, suggesting sufficient data to infer general patterns and conclusions [20]. Finally, we note that we did not apply a multiple testing adjustment to statistical tests performed here, reflecting the exploratory nature of our analyses (hypothesis generating). Future work to replicate findings in other contexts should use a hypothesis-testing framework.

## 5. Conclusions

Here, we introduced a GPS-based approach for quantifying routine activity spaces that summarizes time-weighted exposure to food, physical activity, and environmental contexts beyond the residential neighborhood. Applying this approach to midlife adults in a large, urban county, we found that age and gender were largely unrelated to routine activity space, while differences by race and ethnicity were. Specifically, Black and Hispanic participants spent less time near food and physical activity resources compared to White participants. These findings persisted even after adjustment for population density, poverty, crime, land use, and walkability. In the broader research landscape, these findings corroborate systemic inequities but extend prior research by demonstrating that these inequities are not confined simply to where people live, but also to where people spend their time outside of the home.

This work has both methodological and applied implications. Methodologically, this work underscores the importance of moving beyond static residential measures to dynamic and time-weighted assessments of environmental exposure. After applying these methods, our analysis suggests that structural inequities shape daily mobility patterns as well as access to health-promoting resources. Future research should examine how these disparities translate into differences in health risk, and whether specific domains of activity (e.g., work, commuting, or social travel) disproportionately impact risk. Longitudinal analyses, replication in other geographic settings, and integration with momentary or behavioral data are the necessary next steps to identify causal pathways and to inform future, multi-level interventions.

## Figures and Tables

**Table 1:** Participant characteristics (N = 340).

Characteristics	Count (%) or Mean ± SD
Gender	
Man	135 (39.7)
Woman	200 (58.8)
Transgender or Non-Binary	5 (1.5)
Age	50.4 ± 7.3
Race/Ethnicity	
Non-Hispanic White	123 (36.2)
Non-Hispanic Black	124 (36.5)
Hispanic	93 (27.4)
Education	
Less Than High School	13 (3.8)
High School	93 (27.4)
College—2 to 4 years	124 (36.5)
College—5+ years	110 (32.4)
Marital Status	
Never Married	115 (33.8)
Married	133 (39.1)
Divorced/Separated	85 (25.0)
Widowed	5 (1.5)
Income (household $/year)	
<50,000	95 (27.9)
50,000–150,000	126 (37.1)
>150,000	75 (22.1)
No Answer	44 (12.9)
BMI (kg/m^2^)	29.9 ± 7.2
Missing	3 (0.9)
Walk Score^®^	75.0 ± 17.3

**Table 2:** GPS measured points summary statistics (*n* = 340).

Including Minutes Spent in Home Buffer	Measure	Minimum	Mean ± SD	Maximum
Yes	Total GPS Points (Minutes)	529	21,146.2 ± 7543.1	29,744
	Number of Days GPS Was Used	2	18.4 ± 4.6	21
No	Total GPS Points (Minutes)	28	4173.8 ± 4246.9	27,379
	Number of Days GPS Was Used	1	14.4 ± 5.2	21

**Table 3:** Summary statistics for average closest distances and average kernel density raster values for various location types across all participants (N = 340) ^1^.

Location Type	Minimum	Mean ± SD	Maximum
	Average Closest Distance in Meters		
Public Park	131.5	469.7 ± 257.4	3553.9
Bike Path Segment	34.2	690.8 ± 786.5	12,585.5
Physical Activity Location	89.5	492.4 ± 300.2	3730.1
Pedestrian Segment	9.7	167.1 ± 254.3	3105.5
Public Transit	26.7	344.7 ± 617.1	6493.0
Street Intersection	16.3	41.9 ± 15.6	121.6
Convenience Store	88.7	385.5 ± 205.3	1424.5
Any Fast Food Restaurant	78.3	319.9 ± 236.7	3165.5
Fast Food Chain Restaurant	107.4	412.4 ± 270.2	3292.6
Supermarket	186.1	684.2 ± 338.1	4228.3
Wholesale Club Store	1297.8	3885.6 ± 2012.2	21,782.7
	Average Kernel Density (Per Square Mile) Raster Value		
Public Park	0.7	4.0 ± 1.2	6.5
Bike Path Segment	0.1	2.0 ± 0.9	3.9
Physical Activity Location	0.4	5.3 ± 3.2	14.5
Pedestrian Segment	0.6	59.3 ± 29.4	170.2
Public Transit	0.8	49.1 ± 20.6	114.5
Street Intersection	112.3	500.0 ± 179.6	1258.7
Convenience Store	1.0	5.4 ± 2.0	9.7
Any Fast Food Restaurant	1.1	14.0 ± 7.8	42.0
Fast Food Chain Restaurant	0.7	6.0 ± 2.8	14.4
Supermarket	0.2	1.3 ± 0.5	2.4
Wholesale Club Store	0.0	0.0 ± 0.0	0.1

^1^ All activity space measures for each participant were first averaged across all minute-level measurements in the 21-day study period, not including minutes when they were at home.

**Table 4:** Summary statistics for activity space raster values across all participants (N = 340) ^1^.

Variable (Raster Value)	Minimum	Mean ± SD	Maximum
ACS Hispanic (%)	2.6	21.3 ± 13.5	87.0
ACS Non-Hispanic White (%)	2.0	44.8 ± 17.8	82.1
ACS Non-Hispanic Black (%)	1.4	21.8 ± 20.9	91.6
ACS Non-Hispanic Asian (%)	0.2	8.7 ± 4.5	22.8
ACS Race Entropy	0.2	0.6 ± 0.1	0.8
ACS Female-Headed Households (%)	21.5	37.1 ± 7.2	69.5
ACS Renter Occupied (%)	9.7	50.0 ± 12.0	75.8
ACS Same House as 1 Year Ago (%)	59.9	82.9 ± 5.3	93.1
ACS Moved within 1 Year (%)	6.0	16.6 ± 5.1	30.8
ACS Public Assistance (%)	0.8	2.6 ± 1.1	7.6
ACS Poverty (%)	4.8	14.8 ± 6.3	42.7
ACS Unemployment (%)	1.2	4.5 ± 2.0	12.2
ACS Population Density (number of people per sq km)	2941.9	18,471.4 ± 8740.8	46,134.6
ACS Total Population (number of people)	1337.6	3927.7 ± 742.8	7773.2
EPA National Walkability Index	6.7	14.7 ± 1.3	17.2
Crime Burglary (events/year)	20.5	101.3 ± 40.2	271.7
Crime Murder (events/year)	13.3	211.5 ± 161.6	966.0
Crime Personal (events/year)	19.7	162.6 ± 85.5	551.4
Crime Robbery (events/year)	22.1	248.7 ± 124.3	794.4
Crime Vehicle (events/year)	16.7	134.9 ± 53.0	321.3
Crime Larceny (events/year)	43.8	153.8 ± 49.4	354.1
Crime Property (events/year)	40.4	141.2 ± 38.9	282.8
Crime Rape (events/year)	24.7	117.3 ± 43.3	291.3
Total Crime (events/year)	37.6	144.0 ± 40.5	270.3
Land Use—Commercial	0.0	0.1 ± 0.0	0.3
Land Use—Entropy	0.3	0.4 ± 0.0	0.6
Normalized Difference Vegetation Index, Jan ’20	0.1	0.2 ± 0.0	0.3
Normalized Difference Vegetation Index, June ’20	0.2	0.3 ± 0.1	0.6

^1^ All activity space measures for each participant were first averaged across all minute-level measurements in the 21-day study period, not including minutes when they were at home.

**Table 5:** Difference in means and 95% confidence intervals of average kernel density raster values of location types by participant demographics.

Location Type (Average Kernel Density)	Gender (Male–Female) ^1^	Age (≥50–<50)	Non-Hispanic Black (NH Black–NH White)	Hispanic (Hispanic–NH White)
Public Park	0.365 ** (0.103, 0.626)	−0.132 (−0.392, 0.127)	−0.861 *** (−1.160, −0.562)	−0.414 ** (−0.720, −0.108)
Bike Path Segment	0.252 * (0.050, 0.455)	−0.180 (−0.378, 0.019)	−0.543 *** (−0.774, −0.313)	−0.351 ** (−0.597, −0.104)
Physical Activity Location	1.027 ** (0.329, 1.726)	−0.483 (−1.168, 0.202)	−2.778 *** (−3.545, −2.011)	−1.619 *** (−2.439, −0.800)
Pedestrian Segment	5.586 (−0.772, 11.944)	−3.881 (−10.128, 2.366)	−14.691 *** (−22.144, −7.238)	−7.653 (−15.354, 0.047)
Public Transit	3.813 (−0.628, 8.254)	−2.161 (−6.555, 2.233)	−6.568 * (−11.927, −1.210)	−5.282 (−10.596, 0.032)
Street Intersection	23.957 (−15.398, 63.312)	−16.889 (−55.146, 21.367)	−82.646 *** (−127.761, −37.531)	−46.357 (−93.543, 0.829)
Convenience Store	0.563 * (0.132, 0.994)	−0.426 * (−0.849, −0.004)	−1.449 *** (−1.947, −0.950)	−0.409 (−0.912, 0.094)
Any Fast Food Restaurant	1.961 * (0.244, 3.678)	−0.940 (−2.612, 0.732)	−5.468 *** (−7.417, −3.519)	−2.573 * (−4.558, −0.587)
Fast Food Chain Restaurant	0.649 * (0.049, 1.249)	−0.409 (−0.996, 0.177)	−1.837 *** (−2.524, −1.149)	−0.698 * (−1.393, −0.002)
Supermarket	0.126 * (0.026, 0.226)	−0.108 * (−0.206, −0.010)	−0.373 *** (−0.488, −0.258)	−0.117 * (−0.232, −0.003)
Wholesale Club Store	0.002 (−0.000, 0.004)	−0.002 * (−0.004, −0.000)	−0.006 *** (−0.008, −0.003)	0.002 * (0.001, 0.004)

* *p* < 0.05; ** *p* < 0.01; *** *p* < 0.001. ^1^ Due to the small number of individuals who did not identify as male or female (n = 5), they are excluded from the comparison of males to females.

**Table 6:** Unadjusted correlations and 95% confidence intervals of average kernel density raster values of location types with average raster values of geographic characteristics from the American Community Survey, EPA walkability, crime reporting, and satellite measurements of land use and vegetative index.

Location Type (Average Kernel Density)	ACS Population Density	ACS Poverty	Total Crime	Land Use—Commercial	Normalized Difference Vegetation Index	EPA National Walkability Index
Public Park	0.76 *** (0.69, 0.83)	−0.06 (−0.17, 0.04)	0.40 *** (0.30, 0.50)	0.67 *** (0.59, 0.75)	−0.64 *** (−0.72, −0.56)	0.74 *** (0.66, 0.81)
Bike Path Segment	0.68 *** (0.60, 0.76)	0.04 (−0.06, 0.15)	0.55 *** (0.46, 0.64)	0.64 *** (0.56, 0.73)	−0.71 *** (−0.78, −0.63)	0.63 *** (0.54, 0.71)
Physical Activity Location	0.72 *** (0.65, 0.79)	−0.31 *** (−0.41, −0.21)	0.42 *** (0.32, 0.51)	0.78 *** (0.71, 0.84)	−0.65 *** (−0.73, −0.57)	0.69 *** (0.61, 0.76)
Pedestrian Segment	0.77 *** (0.70, 0.84)	0.03 (−0.08, 0.14)	0.50 *** (0.40, 0.59)	0.66 *** (0.58, 0.74)	−0.69 *** (−0.77, −0.61)	0.59 *** (0.50, 0.68)
Public Transit	0.75 *** (0.68, 0.82)	0.24 *** (0.14, 0.35)	0.63 *** (0.54, 0.71)	0.64 *** (0.56, 0.72)	−0.74 *** (−0.81, −0.66)	0.63 *** (0.55, 0.71)
Street Intersection	0.58 *** (0.50, 0.67)	−0.12 * (−0.23, −0.02)	0.62 *** (0.53, 0.70)	0.81 *** (0.75, 0.88)	−0.70 *** (−0.78, −0.62)	0.66 *** (0.57, 0.74)
Convenience Store	0.75 *** (0.67, 0.82)	−0.15 ** (−0.26, −0.05)	0.44 *** (0.34, 0.53)	0.72 *** (0.64, 0.79)	−0.77 *** (−0.84, −0.70)	0.77 *** (0.70, 0.84)
Any Fast Food Restaurant	0.71 *** (0.63, 0.78)	−0.26 *** (−0.36, −0.15)	0.49 *** (0.40, 0.58)	0.83 *** (0.77, 0.89)	−0.70 *** (−0.78, −0.63)	0.68 *** (0.60, 0.76)
Fast Food Chain Restaurant	0.70 *** (0.62, 0.77)	−0.19 *** (−0.30, −0.09)	0.52 *** (0.43, 0.61)	0.79 *** (0.73, 0.86)	−0.76 *** (−0.83, −0.69)	0.72 *** (0.65, 0.79)
Supermarket	0.70 *** (0.63, 0.78)	−0.19 *** (−0.29, −0.09)	0.40 *** (0.30, 0.50)	0.68 *** (0.61, 0.76)	−0.77 *** (−0.84, −0.70)	0.79 *** (0.72, 0.85)
Wholesale Club Store	0.24 *** (0.13, 0.34)	−0.32 *** (−0.42, −0.22)	−0.07 (−0.17, 0.04)	0.29 *** (0.19, 0.39)	−0.48 *** (−0.58, −0.39)	0.56 *** (0.47, 0.64)

* *p* < 0.05; ** *p* < 0.01; *** *p* < 0.001. Color key: −1.00 to −0.50, −0.50 to −0.20, −0.20 to 0.20, 0.20 to 0.50, 0.50 to 1.00.

**Table 7:** Statistically significant partial correlations ^1^ of average kernel density raster values for location types with the demographic and geographic variables from Table 5 and Table 6.

	Participant Characteristics				Environmental Contexts					
Activity Space (kd)	Male	Age ≥ 50	NH Black	Hispanic	ACS Pop. Density	ACS Poverty	Total Crime	Land Use Com.	NDVI	EPA NWI
Public Park			−0.18 **	−0.11 *	0.50 ***		0.22 ***			0.44 ***
Bike Path Segment			−0.26 ***	−0.19 ***	0.39 ***		0.38 ***		−0.28 ***	
Physical Activity Location			−0.23 ***	−0.22 ***	0.57 ***	−0.54 ***	0.51 ***		−0.21 ***	0.20 ***
Pedestrian Segment			−0.13 *		0.60 ***		0.36 ***		−0.24 ***	
Public Transit			−0.18 ***	−0.12 *	0.59 ***	0.35 ***	0.39 ***	0.19 ***	−0.27 ***	0.19 ***
Street Intersection					0.17 **	−0.25 ***	0.51 ***	0.39 ***	−0.24 ***	0.15 **
Convenience Store			−0.21 ***		0.53 ***	−0.32 ***	0.39 ***		−0.38 ***	0.38 ***
Any Fast Food Restaurant			−0.13 *		0.52 ***	−0.50 ***	0.53 ***	0.26 ***	−0.29 ***	
Fast Food Chain Restaurant			−0.18 ***		0.46 ***	−0.42 ***	0.52 ***	0.15 **	−0.39 ***	0.20 ***
Supermarket			−0.27 ***	−0.18 **	0.44 ***	−0.35 ***	0.35 ***	−0.14 *	−0.43 ***	0.42 ***
Wholesale Club Store					−0.17 **	−0.19 ***	−0.17 **		−0.37 ***	0.36 ***

* *p* < 0.05; ** *p* < 0.01; *** *p* < 0.001. ^1^ Each partial correlation was estimated while controlling for all other demographic and geographic variables.

## Data Availability

The raw data supporting the conclusions of this article will be made available by the authors upon reasonable request.

## Supplementary Materials

The following supporting information can be downloaded at: https://www.mdpi.com/article/10.3390/s26061902/s1, Figure S1: Correlations of Average Closest Distances and Average Raster Values For Location Types Excluding Time Spent At Home. Figure S2: Correlation heatmap of average raster values for American Community Survey, crime, and satellite data, only while away from home. Table S1: Unadjusted correlations and 95% confidence intervals of average kernel density raster values of location types with participant demographics. Table S2: Group means of average kernel density raster values of location types with participant demographics. Table S3: Group means of average kernel density raster values of location types by season. Table S4: Difference of means and 95% confidence intervals of average kernel density raster values of location types by participant demographics including participant time in home buffer. Table S5: Unadjusted correlations and 95% confidence intervals of average kernel density raster values of location types with average raster values of geographic characteristics from the American Community Survey, EPA walkability, crime reporting, and satellite measurements of land use and vegetative index including participant time in home buffer. Table S6: Statistically significant partial correlations1 of average kernel density raster values for location types with the demographic and geographic variables from Tables S3 and S4, including participant time spent in home buffer.

## References

[1] Hales C.M., Carroll M.D., Fryar C.D., Ogden C.L. (2017). Prevalence of Obesity Among Adults and Youth: United States, 2015–2016. NCHS Data Brief. https://www.cdc.gov/nchs/data/databriefs/db288.pdf.

[2] Fuchs M.A., Sato K., Niedzwiecki D., Ye X., Saltz L.B., Mayer R.J., Mowat R.B., Whittom R., Hantel A., Benson A. (2014). Sugar-sweetened beverage intake and cancer recurrence and survival in CALGB 89803 (Alliance). PLoS ONE.

[3] Moore S.C., Lee I.M., Weiderpass E., Campbell P.T., Sampson J.N., Kitahara C.M., Keadle S.K., Arem H., Berrington de Gonzalez A., Hartge P. (2016). Association of Leisure-Time Physical Activity With Risk of 26 Types of Cancer in 1.44 Million Adults. JAMA Intern. Med..

[4] Pool L.R., Ning H., Lloyd-Jones D.M., Allen N.B. (2017). Trends in Racial/Ethnic Disparities in Cardiovascular Health Among US Adults From 1999–2012. J. Am. Heart Assoc..

[5] Gabriel K.P., Sternfeld B., Colvin A., Stewart A., Strotmeyer E.S., Cauley J.A., Dugan S., Karvonen-Gutierrez C. (2017). Physical activity trajectories during midlife and subsequent risk of physical functioning decline in late mid-life: The Study of Women’s Health Across the Nation (SWAN). Prev. Med..

[6] Lachman M.E., Teshale S., Agrigoroaei S. (2014). Midlife as a Pivotal Period in the Life Course: Balancing Growth and Decline at the Crossroads of Youth and Old Age. Int. J. Behav. Dev..

[7] Social Determinants of Health. https://www.cdc.gov/about/priorities/why-is-addressing-sdoh-important.html.

[8] Kärmeniemi M., Lankila T., Ikäheimo T., Koivumaa-Honkanen H., Korpelainen R. (2018). The Built Environment as a Determinant of Physical Activity: A Systematic Review of Longitudinal Studies and Natural Experiments. Ann. Behav. Med. A Publ. Soc. Behav. Med..

[9] Althoff T., Ivanovic B., King A.C., Hicks J.L., Delp S.L., Leskovec J., Althoff T., Ivanovic B., King A.C., Hicks J.L. (2025). Countrywide natural experiment links built environment to physical activity. Nature.

[10] Ferdinand A.O., Sen B., Rahurkar S., Engler S., Menachemi N. (2012). The Relationship Between Built Environments and Physical Activity: A Systematic Review. Am. J. Public Health.

[11] Perchoux C., Chaix B., Cummins S., Kestens Y. (2013). Conceptualization and measurement of environmental exposure in epidemiology: Accounting for activity space related to daily mobility. Health Place.

[12] Siddiqui N.Z., Wei L., Mackenbach J.D., Pinho M.G.M., Helbich M., Schoonmade L.J., Beulens J.W.J., Siddiqui N.Z., Wei L., Mackenbach J.D. (2024). Global positioning system-based food environment exposures, diet-related, and cardiometabolic health outcomes: A systematic review and research agenda. Int. J. Health Geogr..

[13] Liu Y., Kwan M.-P., Yu C. (2023). The uncertain geographic context problem (UGCoP) in measuring people’s exposure to green space using the integrated 3S approach. Urban For. Urban Green..

[14] Thierry B., Chaix B., Kestens Y., Thierry B., Chaix B., Kestens Y. (2013). Detecting activity locations from raw GPS data: A novel kernel-based algorithm. Int. J. Health Geogr..

[15] Jia T., Ji Z. (2017). Understanding the Functionality of Human Activity Hotspots from Their Scaling Pattern Using Trajectory Data. ISPRS Int. J. Geo-Inf..

[16] Hahsler M., Piekenbrock M., Doran D. (2019). dbscan: Fast Density-Based Clustering with R. J. Stat. Softw..

[17] Hirsch J.A., Winters M., Clarke P., McKay H., Hirsch J.A., Winters M., Clarke P., McKay H. (2014). Generating GPS activity spaces that shed light upon the mobility habits of older adults: A descriptive analysis. Int. J. Health Geogr..

[18] Bronas U.G., Kershaw K.N., Tu J., Ryder N., Westra J., Redondo-Sáenz D., Tintle N. (2026). Everyday Environmental Exposures and Mid-Life Dietary and Physical Activity Variations: E3 Study Protocol. Front. Public Health.

[19] Stanley K., Yoo E.-H., Paul T., Bell S. (2018). How many days are enough?: Capturing routine human mobility. Int. J. Geogr. Inf. Sci..

[20] Zenk S.N., Matthews S.A., Kraft A.N., Jones K.K. (2018). How many days of global positioning system (GPS) monitoring do you need to measure activity space environments in health research?. Health Place.

[21] Tintle N., Tu J., Luong A., Min S., Kershaw K.N., Bronas U.G. (2025). Semi-Automated Processing of Harmonized Accelerometer and GPS Data in R: AGPSR. Sensors.

[22] Eisenberg-Guyot J., Moudon A.V., Hurvitz P.M., Mooney S.J., Whitlock K.B., Saelens B.E. (2019). Beyond the bus stop: Where transit users walk. J. Transp. Health.

[23] Jankowska M.M., Natarajan L., Godbole S., Meseck K., Sears D.D., Patterson R.E., Kerr J. (2017). Kernel Density Estimation as a Measure of Environmental Exposure Related to Insulin Resistance in Breast Cancer Survivors. Cancer Epidemiol. Biomark. Prev..

[24] Silverman B. (1986). Density Estimation for Statistics and Data Analysis.

[25] Carr L.J., Dunsiger S.I., Marcus B.H. (2010). Walk Score™ As a Global Estimate of Neighborhood Walkability. Am. J. Prev. Med..

[26] Agurs-Collins T., Alvidrez J., Ferreira S.E., Evans M., Gibbs K., Kowtha B., Pratt C., Reedy J., Shams-White M., Brown A.G. (2024). Perspective: Nutrition Health Disparities Framework: A Model to Advance Health Equity. Adv. Nutr..

[27] McCullough M.L., Chantaprasopsuk S., Islami F., Rees-Punia E., Um C.Y., Wang Y., Leach C.R., Sullivan K.R., Patel A.V. (2022). Association of Socioeconomic and Geographic Factors With Diet Quality in US Adults. JAMA Netw. Open.

[28] Schertz K.E., Saxon J., Cardenas-Iniguez C., Bettencourt L.M.A., Ding Y., Hoffmann H., Berman M.G., Schertz K.E., Saxon J., Cardenas-Iniguez C. (2021). Neighborhood street activity and greenspace usage uniquely contribute to predicting crime. npj Urban Sustain..

[29] Schusler T., Weiss L., Treering D., Balderama E. (2018). Research note: Examining the association between tree canopy, parks and crime in Chicago. Landsc. Urban Plan..

[30] Groff E., McCord E.S., Groff E., McCord E.S. (2011). The role of neighborhood parks as crime generators. Secur. J..

[31] Askey A.P., Taylor R., Groff E., Fingerhut A. (2018). Fast Food Restaurants and Convenience Stores: Using Sales Volume to Explain Crime Patterns in Seattle. Crime Delinq..

[32] Wolfe S.E., Pyrooz D.C. (2014). Rolling Back Prices and Raising Crime Rates? The Walmart Effect on Crime in the United States. Br. J. Criminol..

[33] Singleton C.R., Winata F., Adams A.M., McLafferty S.L., Sheehan K.M., Zenk S.N. (2022). County-level associations between food retailer availability and violent crime rate. BMC Public Health.

