# Supplementary material for: Demographic Associations with GPS-Inferred Routine Activity Spaces: Data from the Everyday Environments and Experiences (E3) Study
Source: Sensors (Basel). 2026 Mar 18;26(6):1902. doi: 10.3390/s26061902 (PMC13030801; doi:10.3390/s26061902)
Supplement: Supplementary file 1 [file sensors-26-01902-s001.zip › sensors-4111671-supplementary.pdf]

Supplemental Tables and Figures

Figure S1: Correlations of Average Closest Distances and Average Raster Values For Location Types Excluding Time Spent At Home

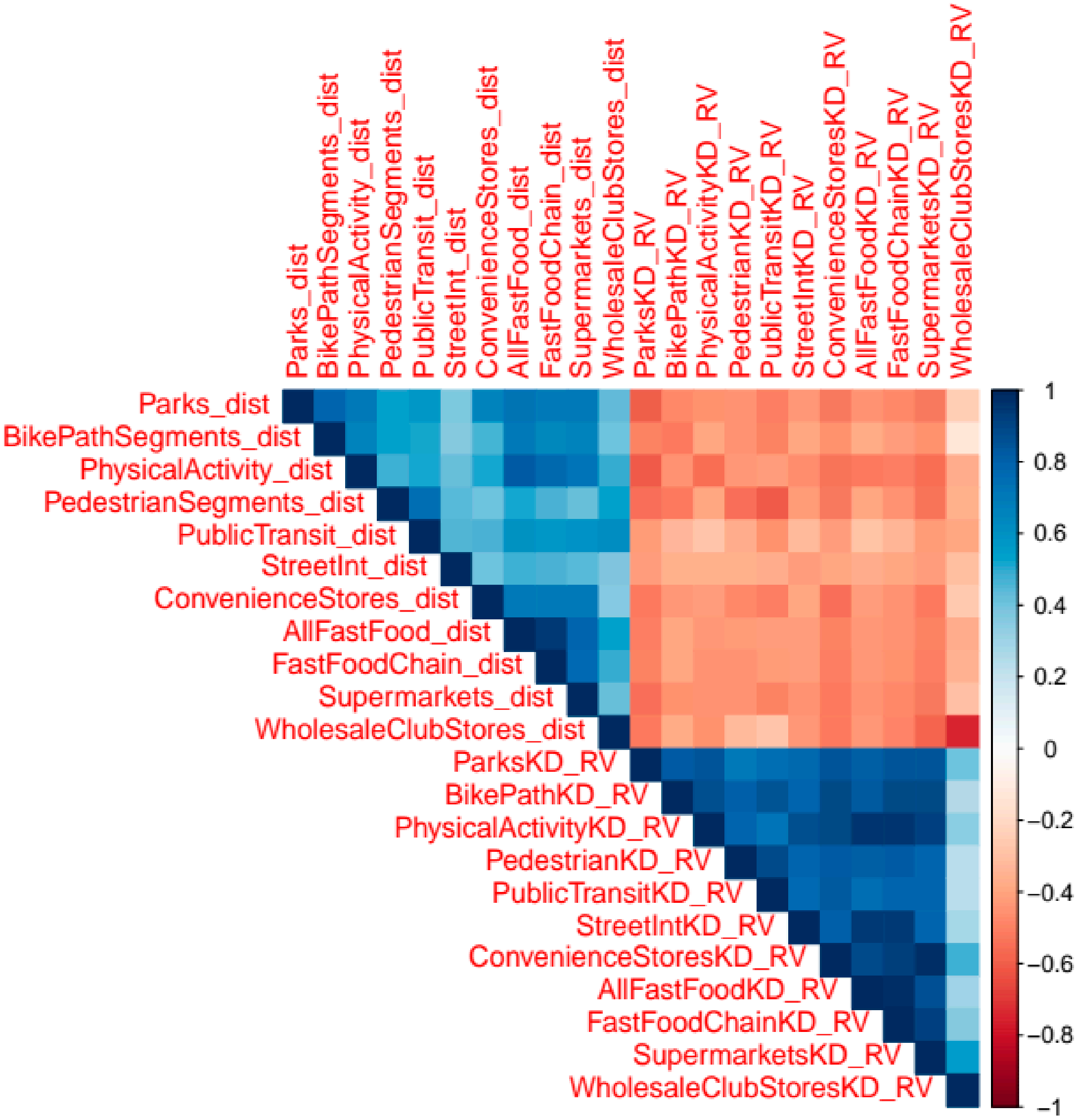

Figure S2: Correlation heatmap of average raster values for American Community Survey, crime, and satellite data, only while away from home.

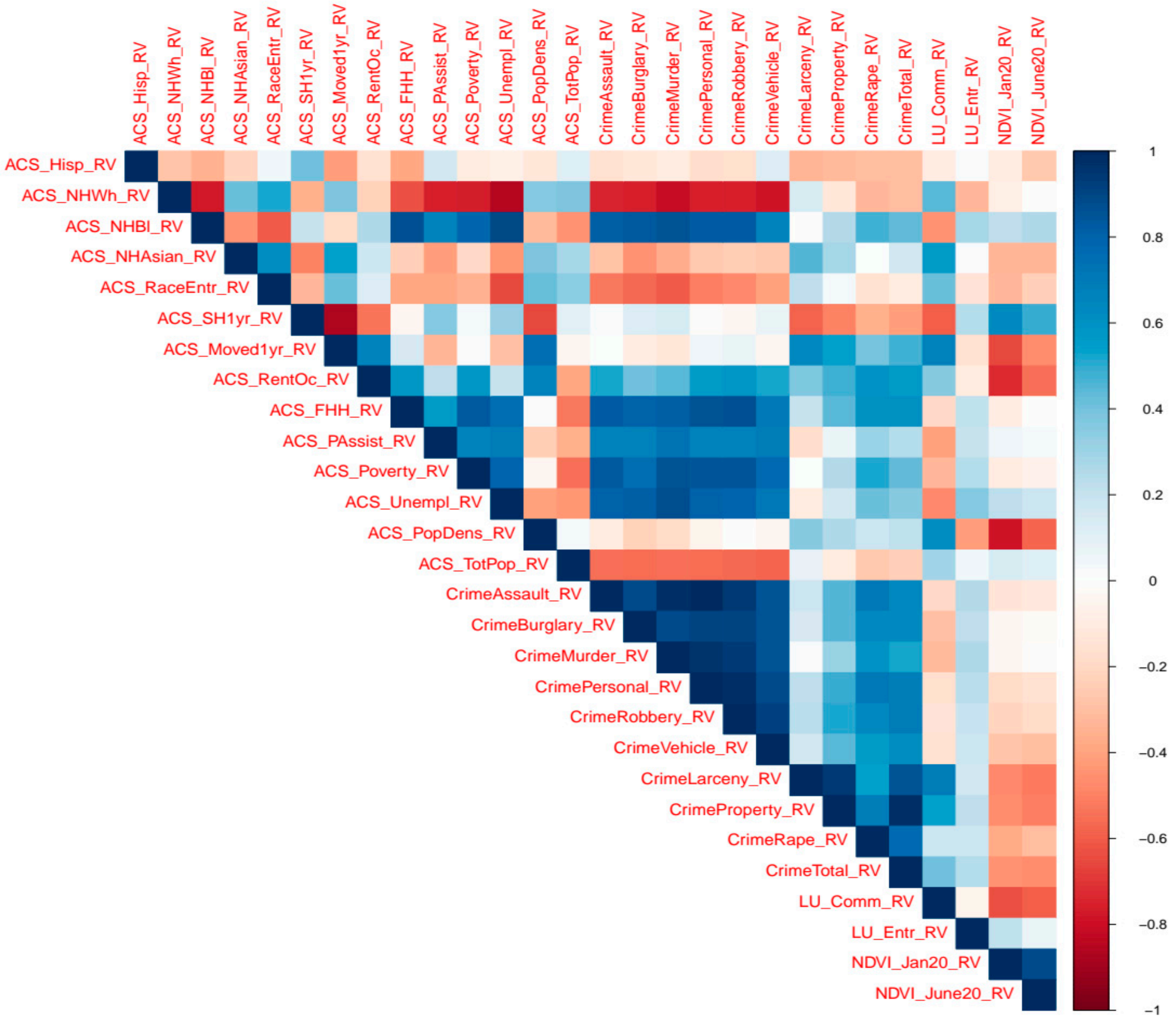

Table S1: Unadjusted correlations and 95% confidence intervals of average kernel density raster values of location types with participant demographics.

**Location Type (Average Kernel Density)**	**Male**	**Age ≥ 50**	**Non-Hispanic Black**	**Non-Hispanic White**	**Hispanic**
Public Park	0.13* (0.02, 0.23)	-0.10 (-0.21, 0.01)	-0.31*** (-0.41, -0.21)	0.30*** (0.19, 0.40)	0.02 (-0.09, 0.12)
Bike Path Segment	0.13* (0.03, 0.24)	-0.08 (-0.19, 0.02)	-0.20*** (-0.30, -0.10)	0.21*** (0.11, 0.32)	-0.01 (-0.12, 0.09)
Physical Activity Location	0.16** (0.06, 0.27)	-0.08 (-0.19, 0.03)	-0.32*** (-0.42, -0.22)	0.35*** (0.25, 0.45)	-0.03 (-0.14, 0.08)
Pedestrian Segment	0.08 (-0.02, 0.19)	-0.03 (-0.14, 0.07)	-0.14* (-0.24, -0.03)	0.08 (-0.03, 0.19)	0.06 (-0.04, 0.17)
Public Transit	0.09 (-0.02, 0.19)	-0.01 (-0.12, 0.09)	-0.07 (-0.18, 0.04)	0.06 (-0.05, 0.17)	0.01 (-0.10, 0.12)
Street Intersection	0.08 (-0.03, 0.18)	-0.10 (-0.21, 0.00)	-0.20*** (-0.31, -0.10)	0.19*** (0.08, 0.29)	0.02 (-0.09, 0.12)
Convenience Store	0.15** (0.05, 0.26)	-0.14* (-0.24, -0.03)	-0.40*** (-0.50, -0.31)	0.26*** (0.16, 0.36)	0.16** (0.05, 0.26)
Any Fast Food Restaurant	0.14* (0.03, 0.24)	-0.13* (-0.24, -0.02)	-0.33*** (-0.43, -0.23)	0.29*** (0.18, 0.39)	0.05 (-0.06, 0.16)
Fast Food Chain Restaurant	0.13* (0.03, 0.24)	-0.14* (-0.24, -0.03)	-0.36*** (-0.46, -0.26)	0.27*** (0.16, 0.37)	0.10 (-0.01, 0.20)
Supermarket	0.14* (0.03, 0.24)	-0.12* (-0.22, -0.01)	-0.40*** (-0.50, -0.31)	0.28*** (0.17, 0.38)	0.14* (0.03, 0.24)
Wholesale Club Store	0.07 (-0.03, 0.18)	-0.13* (-0.24, -0.02)	-0.45*** (-0.55, -0.36)	0.17** (0.07, 0.28)	0.30*** (0.20, 0.40)

Significance indicated by * for p>0.05, ** for p<0.01, * for p<0.001.**

Table S2: Group means of average kernel density raster values of location types with participant demographics.

Location Type (Average Kernel Density)	Male	Female	Age ≥ 50	Age < 50	Non-Hispanic Black	Non-Hispanic White	Hispanic
Public Park	4.18	3.82	3.91	4.04	3.54	4.40	3.99
Bike Path Segment	2.11	1.85	1.87	2.05	1.71	2.26	1.90
Physical Activity Location	5.92	4.90	5.10	5.58	4.01	6.79	5.17
Pedestrian Segment	62.42	56.83	57.35	61.23	52.02	66.71	59.06
Public Transit	51.22	47.41	48.04	50.20	46.37	52.94	47.66
Street Intersection	513.10	489.14	491.66	508.55	460.14	542.78	496.42
Convenience Store	5.70	5.14	5.17	5.59	4.57	6.02	5.61
Any Fast Food Restaurant	15.13	13.17	13.56	14.50	11.25	16.72	14.14
Fast Food Chain Restaurant	6.33	5.68	5.76	6.17	4.98	6.82	6.12
Supermarket	1.41	1.28	1.28	1.39	1.13	1.50	1.38
Wholesale Club Store	0.03	0.03	0.03	0.03	0.03	0.04	0.04

Table S3: Group means of average kernel density raster values of location types by season

Location Type (Average Kernel Density)	Spring (March-May)	Summer (June-August)	Fall (September-November)	Winter (December-February)	ANOVA P-Value
Public Park	4.05	3.86	3.92	4.07	0.63
Bike Path Segment	1.97	1.96	1.93	1.99	0.98
Physical Activity Location	5.32	5.42	5.23	5.37	0.98
Pedestrian Segment	58.92	58.59	58.26	61.10	0.91
Public Transit	49.17	49.42	48.28	49.57	0.98
Street Intersection	489.98	500.49	498.14	509.07	0.93
Convenience Store	5.30	5.37	5.29	5.52	0.86
Any Fast Food Restaurant	13.81	13.97	13.59	14.65	0.82
Fast Food Chain Restaurant	5.85	5.91	5.83	6.21	0.77
Supermarket	1.32	1.34	1.33	1.34	0.99
Wholesale Club Store	0.03	0.03	0.03	0.03	0.86

Table S4: Difference of means and 95% confidence intervals of average kernel density raster values of location types by participant demographics including participant time in home buffer

Location Type (Average Kernel Density)	Gender1 (Male - Female)[1]	Age (≥50 - <50)	Non-Hispanic Black (NH Black - NH White)	Hispanic (Hispanic - NH White)
Public Park	0.381* (0.088, 0.674)	-0.270 (-0.560, 0.020)	-1.093*** (-1.407, -0.779)	-0.494** (-0.858, -0.130)
Bike Path Segment	0.288** (0.071, 0.505)	-0.168 (-0.382, 0.046)	-0.547*** (-0.789, -0.304)	-0.306* (-0.588, -0.024)
Physical Activity Location	1.100** (0.411, 1.790)	-0.520 (-1.204, 0.164)	-2.835*** (-3.589, -2.081)	-1.639*** (-2.495, -0.782)
Pedestrian Segment	5.467 (-0.690, 11.625)	-1.882 (-8.248, 4.485)	-8.602* (-16.609, -0.596)	-0.143 (-7.684, 7.397)
Public Transit	4.029 (-0.391, 8.449)	-0.520 (-5.030, 3.990)	-3.580 (-9.312, 2.151)	-1.300 (-6.490, 3.889)
Street Intersection	26.116 (-5.935, 58.168)	-31.593 (-64.499, 1.314)	-79.589*** (-118.243, -40.936)	-34.234 (-77.056, 8.588)
Convenience Store	0.701** (0.245, 1.157)	-0.578* (-1.029, -0.126)	-1.872*** (-2.385, -1.360)	-0.194 (-0.740, 0.351)
Any Fast Food Restaurant	2.173** (0.660, 3.686)	-1.859* (-3.386, -0.331)	-5.869*** (-7.625, -4.113)	-2.130* (-4.020, -0.240)
Fast Food Chain Restaurant	0.747** (0.190, 1.304)	-0.720* (-1.278, -0.162)	-2.160*** (-2.787, -1.533)	-0.514 (-1.212, 0.185)
Supermarket	0.143** (0.038, 0.249)	-0.115* (-0.219, -0.011)	-0.440*** (-0.557, -0.322)	-0.071 (-0.194, 0.052)
Wholesale Club Store	0.002 (-0.001, 0.005)	-0.003* (-0.006, -0.001)	-0.010*** (-0.013, -0.007)	0.003** (0.001, 0.005)

*p<0.05; **p<0.01; ***p<0.001

[1]Due to the small number of individuals who did not identify as male or female (n=5), they are excluded from the comparison of males to females.

Table S5: Unadjusted correlations and 95% confidence intervals of average kernel density raster values of location types with average raster values of geographic characteristics from the American Community Survey, EPA walkability, crime reporting, and satellite measurements of land use and vegetative index including participant time in home buffer

Location Type (Average Kernel Density)	ACS Population Density	ACS Poverty	Total Crime	Land Use - Commercial	Normalized Difference Vegetation Index	EPA National Walkability Index
Public Park	0.59*** (0.50, 0.67)	-0.09 (-0.20, 0.01)	0.08 (-0.03, 0.18)	0.45*** (0.35, 0.54)	-0.49*** (-0.59, -0.40)	0.45*** (0.36, 0.55)
Bike Path Segment	0.50*** (0.41, 0.59)	0.05 (-0.05, 0.16)	0.31*** (0.21, 0.42)	0.51*** (0.41, 0.60)	-0.62*** (-0.71, -0.54)	0.36*** (0.26, 0.46)
Physical Activity Location	0.51*** (0.41, 0.60)	-0.31*** (-0.42, -0.21)	0.11 (0.00, 0.21)	0.58*** (0.50, 0.67)	-0.51*** (-0.60, -0.41)	0.50*** (0.41, 0.59)
Pedestrian Segment	0.53*** (0.44, 0.62)	0.18*** (0.08, 0.29)	0.33*** (0.23, 0.43)	0.37*** (0.27, 0.47)	-0.67*** (-0.75, -0.59)	0.31*** (0.21, 0.42)
Public Transit	0.54*** (0.45, 0.63)	0.33*** (0.23, 0.43)	0.41*** (0.31, 0.51)	0.37*** (0.27, 0.47)	-0.70*** (-0.77, -0.62)	0.34*** (0.24, 0.44)
Street Intersection	0.44*** (0.35, 0.54)	-0.10 (-0.21, 0.01)	0.26*** (0.16, 0.36)	0.55*** (0.46, 0.64)	-0.59*** (-0.67, -0.50)	0.47*** (0.37, 0.56)
Convenience Store	0.56*** (0.48, 0.65)	-0.20*** (-0.30, -0.10)	0.09 (-0.02, 0.20)	0.53*** (0.44, 0.62)	-0.71*** (-0.78, -0.63)	0.56*** (0.47, 0.65)
Any Fast Food Restaurant	0.55*** (0.46, 0.64)	-0.28*** (-0.39, -0.18)	0.11 (-0.00, 0.21)	0.60*** (0.52, 0.69)	-0.58*** (-0.67, -0.49)	0.50*** (0.40, 0.59)
Fast Food Chain Restaurant	0.53*** (0.44, 0.62)	-0.25*** (-0.36, -0.15)	0.13* (0.02, 0.23)	0.60*** (0.51, 0.68)	-0.66*** (-0.74, -0.58)	0.53*** (0.44, 0.62)
Supermarket	0.49*** (0.40, 0.59)	-0.24*** (-0.35, -0.14)	0.09 (-0.02, 0.20)	0.54*** (0.45, 0.63)	-0.69*** (-0.77, -0.61)	0.59*** (0.50, 0.68)
Wholesale Club Store	0.16** (0.05, 0.26)	-0.36*** (-0.46, -0.26)	-0.21*** (-0.31, -0.10)	0.19*** (0.08, 0.29)	-0.39*** (-0.48, -0.29)	0.49*** (0.40, 0.59)

*p<0.05; **p<0.01; ***p<0.001

Table S6: Statistically significant partial correlations[1] of average kernel density raster values for location types with the demographic and geographic variables from Tables S3 and S4, including participant time spent in home buffer

	**Participant characteristics**				**Environmental contexts**					
**Activity Space (kd)**	**Male**	**Age ≥ 50**	**NH Black**	**Hispanic**	**ACS Pop. Density**	**ACS Poverty**	**Total Crime**	**Land Use Com.**	**NDVI**	**EPA NWI**
Public Park			-0.23 ***	-0.14 *	0.43 ***		0.19 ***			0.25 ***
Bike Path Segment			-0.22 ***	-0.18 ***	0.25 ***		0.34 ***	0.21 ***	-0.29 ***	0.21 ***
Physical Activity Location			-0.24 ***	-0.20 ***	0.39 ***	-0.45 ***	0.41 ***	0.22 ***		0.21 ***
Pedestrian Segment			-0.12 *		0.29 ***		0.26 ***		-0.37 ***	
Public Transit		0.11 *		-0.13 *	0.29 ***	0.24 ***	0.23 ***		-0.42 ***	0.17 **
Street Intersection			-0.11 *		0.21 ***	-0.23 ***	0.33 ***	0.23 ***	-0.23 ***	0.17 **
Convenience Store			-0.26 ***		0.34 ***	-0.32 ***	0.28 ***		-0.41 ***	0.25 ***
Any Fast Food Restaurant			-0.18 ***		0.40 ***	-0.43 ***	0.38 ***	0.22 ***	-0.20 ***	0.18 **
Fast Food Chain Restaurant			-0.21 ***		0.33 ***	-0.42 ***	0.37 ***	0.19 ***	-0.35 ***	0.19 ***
Supermarket			-0.27 ***		0.23 ***	-0.36 ***	0.29 ***		-0.42 ***	0.29 ***
Wholesale Club Store			-0.17 **			-0.24 ***		-0.17 **	-0.28 ***	0.31 ***

*p<0.05; **p<0.01; ***p<0.001

Each partial correlation was estimated while controlling for all other demographic and geographic variables.
